# Taking ownership of your career: professional development through experiential learning

**DOI:** 10.1186/s12919-021-00211-w

**Published:** 2021-06-22

**Authors:** Verónica A. Segarra, William A. Gentry

**Affiliations:** 1 Department of Biology, High Point University, High Point, NC 27268 USA; 2 Career and Professional Development, High Point University, High Point, NC 27268 USA

**Keywords:** Professional development for scientists, Tenure-track faculty, Tenure, Professoriate, Professional development practicum, Professional development experiential learning, Accomplishing Career Transitions, American Society for Cell Biology, Minorities Affairs Committee

## Abstract

Experiential learning can facilitate the development of transferrable skills necessary for success in attaining tenure and promotion in academia. In this article, we discuss the benefits of designing and implementing an individualized professional development experience or practicum. By doing this, we describe the experiential learning component of the Accomplishing Career Transitions (ACT) Program of the American Society for Cell Biology. The ACT program aims to assist postdoctoral trainees and junior faculty from backgrounds underrepresented in STEM as they strive to transition into tenure-track positions and ultimately attain tenure at research-intensive or teaching-intensive academic institutions.

**Supplementary Information:**

The online version contains supplementary material available at 10.1186/s12919-021-00211-w.

## Background

Some of the transferrable skills that are key for success in the scientific workforce are acquired during academic training at the graduate level [1, 2]. However, supplemental professional development opportunities are needed to sharpen additional competencies, including the ability to set a vision and goals, time management, the ability to work on a team, the ability to collaborate outside the organization, the ability to manage others, and skills in career planning and self-awareness [1, 2]. Many of these additional skills are indispensable for success as a tenure-track faculty member and for the achievement of tenure. These gaps in academic training may be particularly applicable to scientists from backgrounds underrepresented in STEM, who generally have less access to mentoring resources than their peers from well-represented groups [3]. Recognition of this phenomenon is one of the motivations driving the creation and implementation of professional development programs that aim to close these gaps at the postdoctoral and junior faculty level [4–9]. The Accomplishing Career Transitions Program (ACT; https://www.ascb.org/career-development/2021-accomplishing-career-transitions-act-program/) and its summer workshop, in part chronicled in this *BMC Proceedings* Supplement, are examples of programming developed by the American Society for Cell Biology (ASCB).

The program aims to catalyze the successful transition of postdoctoral trainees and junior faculty from backgrounds underrepresented in STEM into tenure-track and ultimately tenured positions at research-intensive or teaching-intensive academic institutions. ASCB ACT is characterized by its longitudinal (two-year), cohort (sustained group interactions), and experiential (practicum) approaches to professional development. Experiential approaches to professional development provide trainees with opportunities to further develop transferrable skills and increase self-efficacy in contexts that simulate potential future work environments [10]. The experiential approach of ASCB’s ACT program works with trainees to develop a series of learning experiences that serve as an individualized professional development practicum. Prior to the development and implementation of this practicum, trainees attend an in-person summer kick-off event that uses case study-based activities, workshops, and panel discussions to introduce many of the basic skills needed to secure a tenure-track academic position and attain tenure (see Table 1 for list and examples of these skills). After integrating this knowledge, trainees are asked to design and implement a series of learning experiences that will help them fill gaps in their professional development.

**Table 1:** Skills often needed to successfully transition into tenured faculty positions

Skill or competency	Specific examples
Writing	Crafting scientific papers, grants, and patent applications, preparing a tenure portfolio
Specialized Technical/ Scientific knowledge base	Performing microscopy or bioinformatic analysis
Interviewing	Presenting oneself as a candidate for a position, in-person or remote seminars, chalk talks, and interviews
Negotiating	Negotiating a job offer, reaching compromises with vendors and collaborators
Setting up, administering, and maintaining a research laboratory	Drafting a budget, selecting and maintaining laboratory equipment, managing shared equipment, hiring and onboarding of trainees/staff, scientific training and supervision of a lab and its members, establishment of standard operating procedures and protocols including processes for data storage and curation of relevant materials
Mentoring	Mentor trainees achieve their full potential and goals in an inclusive and nurturing environment, learn to seek help and cultivate mentors from whom to receive advice (be a mentee)
Managing Conflict	Navigating the conflicts that arise in day to day interactions while working with a team of trainees and/or collaborators
Managing Time	Prioritizing tasks according to urgency and potential for positive impact
Managing collaborations	Establishing and nurturing collaborations to maximize chances for success
Teaching	Learning to establish effective learning communities within undergraduate and graduate courses
Professional Branding: Cultivating a reputation or niche	Selecting important scientific questions to address, disseminating your work to colleagues and impacting the larger field of research
Developing/sustaining a strong network of mentors (including near-peer and peer-mentors)	Strategically recruiting colleagues to serve as sounding boards throughout your career trajectory

In this article, we summarize and discuss the benefits of empowering individual trainees to take charge of their own professional development through experiential learning (practicum) design and implementation. We anchor this discussion in relevant literature focused on contemporary professional development perspectives, while also providing practical strategies and ideas to strengthen common skills that are key for successful transition into tenure-track positions and achievement of tenure. Our hope is that trainees exploring the rest of this supplement and its different articles will gain awareness of the opportunity to design a practicum or series of experiences to help refine and fine-tune those skills. At the conclusion of this article, we provide a guide and resources to design and implement an individualized practicum experience.

## Main text

### Effective active career management: placing the individual at the center of decision-making

Contemporary theories on career management, such as boundaryless and protean career perspectives, place the individual (rather than the university or organization) as the central figure in the management and trajectory of one’s own career [11–13]. It is the responsibility not of the institution but rather of the employee (i.e., postdoc or faculty member) to be proactive in setting individual career goals, whether they be acquiring relevant skills, promotions, higher salaries, or other desired subjective and objective career outcomes [14, 15]. There is no one single approved or typical way to do this; the different pathways and trajectories and ways to manage one’s career are limitless and often highly individualized.

The following sections will discuss the literature supporting three steps that a postdoc or faculty member should pursue in being an active participant in their own career management: needs assessment; goal setting; and creating a development plan and putting the plan into action. This strategy is synergistic with individual development plan (IDP) resources developed by others, including the myIDP Science Careers resource [16]. IDPs and similar strength, weaknesses, opportunities, and threats (SWOT) analyses have previously been reported as successful elements in the theoretical framework of professional development programs [17].

### Needs assessment

The first step a postdoc or faculty member should take is a needs assessment, using their desired academic context and goals as references. For example, while research-intensive and teaching-intensive academic institutions exhibit similarities in the skills needed for success in faculty positions, they also have some key differences. Individuals who are in the process of choosing between a research-intensive or teaching-intensive context can use a combination of informational interviews and guest visits to help them define the academic context that would fit best with their interests as scholars and scientists.

Traditionally, organizations have used a needs assessment to identify training or development needs of their employees [18], with a primary focus on the relevance and perceived utility of the content to be learned [19, 20]. Organizations generally perform this assessment by identifying the business need, performing a gap analysis (to better understand the gap by assessing the current skill or performance levels of employees and comparing them to desired levels) and then choosing the right option to fill that gap.

In modern-day workplaces where the responsibility for career management rests with individuals, the postdocs or faculty members should conduct their own needs analysis and ascertain what gap exactly they need to fill in order to transition successfully to a tenured faculty position. They should first self-assess what their strengths are and what developmental needs they have, whether those are technical in nature or in more person-focused areas such as interviewing, conflict management, or branding. After a self-assessment, the postdocs or faculty members should also solicit insights and opinions from others whom they know well. Having observers like family, friends, peers, and former or current mentors or supervisors will help the postdoc or faculty member get a more well-rounded view of strengths and shortcomings.

After perceiving several shortcomings or developmental needs, it is a best practice to focus on developing one area at a time in order to concentrate efforts on improvement in that area without spreading oneself too thin [21]. The ability to change and develop is maximized by clear focus on one goal that the postdoc or faculty member feels energetic and passionate about, with a clear vision that the change or development will positively benefit themselves and others [21]. The individual must also cultivate the mindset that although this process of development will be challenging and will require time and resources, it is not discouraging or insurmountable.

### Goal setting

Once the postdoc or faculty member has identified the skill or competency that must be developed, the next step is to set a goal. The goal can be behavioral (changing the way one acts), competency-based (improving a specific skill), or outcome-based (focused on meeting a target) [21]. The contemporary model in which individuals rather than organizations set goals for career development [22] has been reinforced by recent research showing that self-set goals are associated with enthusiasm and lower emotional exhaustion, while organization-set goals are associated with anxiety and higher emotional exhaustion [23]. Other research suggests that self-set goals may also be perceived as more attainable due to individuals’ sense of control over the process of choosing their own goals rather than having the goals chosen or set for them [24–26].

Goal setting theory also indicates that the motivation to attain goals is maximized when a person sets both short-term and long-term goals [27, 28]. A long-term goal should be broken down into several short-term goals that function as more achievable steps in pursuit of the longer-term goal [28, 29]. In addition, to maximize one’s chances of goal achievement, the goal should be specific, beneficial to the person, challenging and difficult, yet attainable [30, 31].

### Creating a development plan and putting it into action

Goal-setting should be followed by creating a development plan to achieve it, including four key components: tactics, resources, tracking, and celebrating [21]. Tactics refer to the exact actions one must perform to accomplish a stated goal. Resources are the time and money necessary to attain that goal, as well as the people who will provide support and feedback, and act as accountability partners. These can include one’s boss or supervisor, mentors and peers within the university and colleagues outside of the university. Tracking refers to consistent and frequent checking on one’s progress toward goal attainment. Celebrating refers to ways in which the person will appropriately recognize progress toward goal attainment and commemorate that progress in a meaningful way that is appropriate for that individual.

### Example

Oftentimes, postdocs and pre-tenure faculty need certain skills to be developed or be considered strengths before they can be perceived as ready to successfully transition into tenured faculty positions or be considered for tenure. The following describes a scenario in which a postdoc takes ownership of her own career management, based on the previous steps mentioned. The steps could be applicable for a pre-tenure faculty who is going up for tenure as well. The postdoc received feedback from others that she needs to improve herself in order to be fully considered for tenured positions. However, this feedback was quite general and did not include a clear directive about what areas she needs to improve in specifically. She first performs a needs assessment, using Table 1. Although this table is not an exhaustive list, it does provide several different skills, competencies, and behaviors needed for one to transition successfully into faculty positions and attain tenure. The postdoc sets aside time to perform an honest self-assessment of her perceived strengths and weaknesses, as well as gaps in skills, experience and knowledge. Thinking back on other positions she has applied for in the past, she feels she has been unprepared for interviews and lacks the poise and confidence that other more-seasoned interviewees and applicants seem to naturally possess. She then asks her current supervisor, friends from her time in graduate school, family members, and current peers what they think are her key areas for improvement. Their feedback also identifies interviewing skills as a potential area of improvement. Because of her life-long dream to be a professor, she has the energy and the passion to develop her interviewing skills and sees this as an opportunity that will positively benefit her future. She understands that this is a challenge that will take time and effort, yet is not so difficult that it will leave her frustrated, angry, and discouraged.

In order to set a specific goal and articulate its relevance to her career, she writes down; “Within the next 30 days, I will improve my interviewing skills which will enhance the perception that I am confident, thereby increasing my chances of attaining a faculty position.” With the long-term goal set, she now thinks about short-term goals that can build toward that long-term goal. These short-term goals include learning more about her nonverbal behaviors, particularly around the importance of eye contact, pace of speech, and posture. Another short-term goal is to solicit advice on how she can better her skills from others whom she feels have a commanding presence in a room during interviews or seminars.

Finally, she crafts and implements her plan, making a checklist that clearly lists the tactics she will perform over the next 30 days and enables her to easily keep track of progress. In the first week, she will read a book on how to improve interviewing skills. In the second week, she will take LinkedIn Learning courses on interviewing skills. In the third week, she will observe herself answering questions by talking into a mirror, and will ask a friend to play the role of an interviewer and video her answering questions with her smart phone. Both the postdoc and her friend will review the video and identify strengths and weaknesses, particularly around eye contact, pace of speech, and posture. In addition, she will ask advice on public speaking and how to command a room from her supervisor, who has received rave reviews from attendees in talks and lectures. In the final week, she will schedule “mock interviews” with members of the career services offices of both her current university and her undergraduate alma mater, as this service is free to her as a trainee at her current university and as an alum of her undergraduate university. Her friend, supervisor, and career services are valuable resources that will provide valuable feedback and hold her accountable. After the 30 days, she celebrates with her friends by having a small party in her home, and telling them what she has learned and how she will utilize her new-found skills in her next interview.

### Developing an individualized practicum experience to address gaps in professional development

As part of the ASCB ACT program, we have developed tools that postdocs and junior faculty members can use to develop a customized experiential learning plan or practicum to strengthen gaps in professional development. These tools are designed for individuals who want to engineer their own professional development experience or who train at institutions that do not have professional development program offerings that are appropriate for their needs [32].

As a starting point, individuals can review common skills required for faculty positions (Table 1) and activities to develop them (Table 2). Individuals can then utilize the *ACT Practicum Worksheet* (Additional file 1) to articulate their own needs and goals for professional development and to identify activities that will help them further develop these areas and their timelines, as well as peers and mentors to assist in the implementation of practicum activities and serve as accountability partners. Once the individual has identified areas to focus on, it can be greatly beneficial to describe their planned practicum in the form of a short written proposal, in order to share it with relevant mentors and peers who can provide feedback. Additional file 2 describes the recommended sections for the practicum proposal. Once mentor and peer suggestions have been incorporated into the practicum plan, the individual can begin to implement it. Table 2 lists resources and tools such as feedback from mentors, mock interviews, informational interviews, networking, and program offerings at local Centers of Teaching and Learning, most of which are free of cost. If funds are needed for practicum implementation, individuals can use material generated for practicum proposal and funds received from the ASCB ACT program to advocate for the additional funds required from their home institution.

**Table 2:** Resources and tools that are often available and can be leveraged to develop and refine skills needed to successfully transition into tenured faculty positions. Professional development courses or workshops, as mentioned in the table, are often available through a variety of sources, including home academic institutions, scientific societies, and/or research institutes

Skill or Competency	Resources and Tools
Writing	Professional development courses/workshops, opportunities to practice and receive feedback from mentors, colleagues, and/or collaborators
Specialized Technical/ Scientific knowledge base	Technical courses or collaborations that provide learning and networking opportunities with experts in the field
Interviewing	Mock interviews with mentors and colleagues, informational interviews with those who have recently interviewed for positions
Negotiating	Case-study based professional development courses/workshops, low-stakes opportunities to practice negotiation, informational interviews with colleagues who have recently negotiated faculty position offers
Setting up, administering, and maintaining a research laboratory	Professional development courses/workshops, informational interviews with faculty running effective lab environments, preparing resources such as equipment lists with the feedback of mentors or colleagues
Mentoring	Professional development courses/workshops, opportunities to practice necessary skills inside and outside of the lab
Managing Conflict	Case-study based professional development courses and workshops, opportunities to practice conflict resolution in low-stakes situations or with the help of mentors
Managing Time	Professional development courses/workshops, references describing common strategies for prioritization and avoiding time sinks
Managing collaborations	Case study-based professional development courses/workshops, low-stakes opportunities to practice collaborating with trusted mentors
Teaching	Courses or certifications available at local Centers of Teaching and Learning, opportunities to serve as guest speaker, invited lecturer or instructor of record, creating a course or workshop to meet a learning need identified in the relevant student population
Professional Branding: Cultivating a reputation or niche	Disseminating work through networking, interactions, and presentations at home institutions, relevant scientific conferences or invited talks, and social media/online website presence
Developing/sustaining a strong network of mentors (including near-peer and peer-mentors)	Networking at one’s home institution, at relevant scientific conferences, and through social media

The *ACT Check-in Template* (Additional file 3) is a tool developed to facilitate the important step of establishing a system for regular feedback from others. Postdocs and junior faculty members can complete this form at regular intervals during the implementation of their practicum, then share it with mentors and accountability partners to ask for their feedback. Once the practicum has been completed, it is important to reflect on the career development experiences [10]. To facilitate this reflection, we provide an assessment rubric for individuals to use as a guide (Additional file 4).

### Professional development during the COVID-19 pandemic

During the COVID-19 pandemic, conferences and scientific events including professional development program offerings have been cancelled or shifted to online or remote formats, likely altering the benefits individuals receive from these experiences. While these shifts to online events have often allowed access without the need for travel and with reduced or waived registration fees, the transformational potential of these programs is likely reduced relative to in-person events. While remote formats can replicate passive modes of professional development programming such as traditional lectures, seminars, and career panels, the powerful experiential components of these events, such as opportunities to engage in peer- and near-peer mentoring relationships and networking, are more limited and less engaging. With institutions limited in the types of professional development programming they can offer to their trainees, the design of a professional development practicum by the individual to strategically strengthen a particular set of skills, as described above, might represent an ideal alternative way to facilitate engaging and impactful professional development opportunities in these challenging times.

## Conclusions

The implementation of a professional development practicum, as described in this article, highlights the power of contemporary models of career management that place the individual as the driving force behind their own career trajectory [11–13]. As a result, there is no one single way to implement a practicum experience, rather the individual identifies areas of need such as writing and/or mentoring and chooses from a number or resources and tools to refine those. This article shares strategies and exercises that postdocs and junior faculty can use to assess needs and identify practicum components to address them.

## Supplementary Information

**Additional file 1.** ACT Practicum Worksheet, Microsoft Word worksheet intended to guide your practicum design and implementation process.

**Additional file 2.** ACT Practicum Proposal Sections, Microsoft Word document that lists the recommended sections for a practicum proposal.

**Additional file 3.** ACT Practicum Check-in Template, Microsoft Word document that serves as a guide to evaluate the progress made towards practicum completion.

**Additional file 4.** ACT Practicum Reflection and Assessment, Microsoft Word document containing a rubric to facilitate practicum reflection and assessment upon its completion.

## Data Availability

Not a data-based article.

## Abbreviations

ACT: Accomplishing Career Transitions
ASCB: American Society for Cell Biology
SWOT: Strengths, Weaknesses, Opportunities, and Threats
