# Supplementary material for: Taking ownership of your career: professional development through experiential learning
Source: BMC Proc. 2021 Jun 22;15(Suppl 2):5. doi: 10.1186/s12919-021-00211-w (PMC8217969; doi:10.1186/s12919-021-00211-w)
Supplement: Supplementary file 1 — Additional file 1. ACT Practicum Worksheet, Microsoft Word worksheet intended to guide your practicum design and implementation process. [file 12919_2021_211_MOESM1_ESM.docx]

**Supplemental Materials, Appendix 1: ACT Practicum Worksheet**

Name: Date:

This worksheet is intended to guide your practicum design and implementation process. Be thoughtful in your responses, reflecting on long-term goals and practical applications of practicum activities and outcomes.

1. What are your current career goals?
2. What experiences or skills do you need to have or strengthen to succeed in that career?
3. Through what specific activities or actions might you obtain those experiences or skills?

| **Experience/Skill** | **Activity/Action to Obtain It** |
| --- | --- |
|  |  |
|  |  |
|  |  |
|  |  |
|  |  |

1. How long should activities take to complete successfully? What would they focus on/what content would they cover? How could their quality be assessed?

| **Activity/Action** | **Duration, Focus/Content, Quality** |
| --- | --- |
|  |  |
|  |  |
|  |  |
|  |  |
|  |  |

(continued on next page)

1. Thinking in 3-month increments, how might you structure your activity timeline to obtain desired experiences or skills?

| **Time Period** | **Activity** | **Purpose/Outcome** |
| --- | --- | --- |
|  |  |  |
|  |  |  |
|  |  |  |
|  |  |  |
|  |  |  |

1. How would you know if you are successful? What are potential markers/measures of whether you gained these experiences or skills?

| **Experience/Skill** | **Measure of Success/Obtainment** |
| --- | --- |
|  |  |
|  |  |
|  |  |

1. Who else in the cohort is working in similar areas whom you might wish to collaborate with?

| **Name** | **Focus** | **Institution/email** |
| --- | --- | --- |
|  |  |  |
|  |  |  |
|  |  |  |
|  |  |  |

1. What do you need to do in the next 3 months to get your practicum started? What do you predict accomplishing before the first quarterly check-in?
