# Supplementary material for: Taking ownership of your career: professional development through experiential learning
Source: BMC Proc. 2021 Jun 22;15(Suppl 2):5. doi: 10.1186/s12919-021-00211-w (PMC8217969; doi:10.1186/s12919-021-00211-w)
Supplement: Supplementary file 2 — Additional file 2. ACT Practicum Proposal Sections, Microsoft Word document that lists the recommended sections for a practicum proposal. [file 12919_2021_211_MOESM2_ESM.docx]

**Supplemental Materials, Appendix 2: ACT Practicum Proposal Sections**

**Title**

**Abstract**

**Relevant publications and experiences** (Specify ONLY relevant publications and experiences as it relates to your proposed practicum goals and briefly describe their relevance to proposed Practicum Objectives and Practicum Proposed Methods/Activities. Limit content to one page if possible.)

**Statement of the professional development gaps that the proposed practicum is designed to address** (Provide a summary of your proposed practicum. State succinctly the fundamental gaps or skills it is designed to address. List how the specific goals to be accomplished through the practicum activities address these gaps. To facilitate the design of your goals use the “Practicum worksheet” (Appendix 1), “Check-in Template” (Appendix 3), and “Practicum rubric” (Appendix 4). Try to limit content to one page.)

**Implementation and assessment plan** (Outline your practicum approach to address a gap in your professional development and its feasibility, providing details sufficient to support feasibility (include proposed or tentative timeline of activities). Point out innovative features, and relate them to previous work/experiences by including pertinent references. Indicate how this plan will contribute to your professional development and long-term goals. Your plan must include an assessment strategy and list any resources and mentoring needed from mentors and peers. To facilitate the design of your timeline, goals, and assessment strategy please use the following documents provided to “Practicum worksheet” (Appendix 1), “Check-in Template” (Appendix 3), and “Practicum rubric” (Appendix 4). Do not use more than two pages.)

**Budget** (Design an itemized budget to implement your practicum. List budget items by categories (e.g., travel, meals, supplies, fees, etc.) and include cost-share, if applicable.)

**List of references cited**
