# Supplementary material for: Taking ownership of your career: professional development through experiential learning
Source: BMC Proc. 2021 Jun 22;15(Suppl 2):5. doi: 10.1186/s12919-021-00211-w (PMC8217969; doi:10.1186/s12919-021-00211-w)
Supplement: Supplementary file 3 — Additional file 3. ACT Practicum Check-in Template, Microsoft Word document that serves as a guide to evaluate the progress made towards practicum completion. [file 12919_2021_211_MOESM3_ESM.docx]

**Supplemental Materials, Appendix 3: ACT Practicum Check-in Template**

**Name**:

**Date**:

**Practicum Title:**

**Practicum Goals:**

**Updates from the Last 3 Months:**

1. [Bullet point your project updates or news from the prior 3 months here; What have you worked on and what have you finished? Have you discovered anything new relevant to your practicum? Conclude with a comment on whether you feel like you are ahead of the planned schedule, on schedule, or behind schedule]

**Goals/Plans/Activities for the Next 3 Months:**

1. [Bullet point the plans for the next 3 months, which you will report on in the next check-in]

**Pending:**

1. [Bullet point any pending activities here that do not have updates, with the status of each and/or what they are waiting on]
