# Supplementary material for: Taking ownership of your career: professional development through experiential learning
Source: BMC Proc. 2021 Jun 22;15(Suppl 2):5. doi: 10.1186/s12919-021-00211-w (PMC8217969; doi:10.1186/s12919-021-00211-w)
Supplement: Supplementary file 4 — Additional file 4. ACT Practicum Reflection and Assessment, Microsoft Word document containing a rubric to facilitate practicum reflection and assessment upon its completion. [file 12919_2021_211_MOESM4_ESM.docx]

**Supplemental Materials, Appendix 4: ACT Practicum Reflection and Assessment**

| **Dimension of merit for meeting implementation fidelity threshold** | **Proposed Activity 1** | **Proposed Activity 2** |
| --- | --- | --- |
| **Timing**:  The activity was implemented within the proposed time or with only minor delays. | Planned timeline: Actual timeline:  Assessment: | Planned timeline: Actual timeline:  Assessment: |
| **Duration**:  The activity was not interrupted and achieved its intended duration. | Planned duration: Actual duration:  Assessment: | Planned duration: Actual duration:  Assessment: |
| **Content**:  The activity was implemented with appropriate focus/ content, which aligned with the goals and was responsive to any contextual needs that arose. | Planned content: Actual content:  Assessment: | Planned content: Actual content:  Assessment: |
| **Quality:** The activity was of high enough quality to support attainment of goals. | Assessment: | Assessment: |
| **People:**  As relevant, appropriate people were recruited to participate. | Planned people: Actual people:  Assessment: | Planned people: Actual people:  Assessment: |
